# Art education participation and self-actualization among Chinese college students: the mediating role of creative self-efficacy and the moderating role of openness to experience

**DOI:** 10.3389/fpsyg.2026.1974195

**Published:** 2026-10-14

**Authors:** Kaidi Ma, Jingbo Sun

**Affiliations:** Namseoul University, Cheonan, Chungcheong, Republic of Korea

**Keywords:** art education, China, college students, creative self-efficacy, moderated mediation, openness to experience, self-actualization, wellbeing

## Abstract

Self-actualization, the full use and development of one's talents and potentials, is a central aim of humanistic conceptions of wellbeing, and universities are increasingly expected to serve as meaningful contexts that nurture such growth. Art education has been proposed as one vehicle for this mission, but the psychological mechanisms linking art education participation to self-actualization remain poorly understood. Drawing on self-efficacy theory and self-actualization theory, this cross-sectional study examined whether creative self-efficacy mediates the relationship between art education participation and self-actualization, and whether openness to experience moderates this indirect pathway. A total of 671 Chinese college students (54.8% women; *M* age = 19.9 years, SD = 1.3) from three universities in Shandong Province completed measures of art education participation (the self-reported frequency of involvement in five university art domains), creative self-efficacy, self-actualization, and openness to experience. Correlational analyses showed that art education participation was positively associated with creative self-efficacy (*r* =.52) and self-actualization (*r* =.38). Moderated mediation analysis (PROCESS Model 7, 5,000 bootstrap samples) revealed that creative self-efficacy mediated the effect of art education participation on self-actualization [indirect effect = 0.172, 95% CI (0.132, 0.214), 47.8% of the total effect], and that openness to experience moderated the first stage of this pathway (interaction = 0.09, *p* = 0.005); the index of moderated mediation was significant [0.040, 95% CI (0.008, 0.076)], with the indirect effect increasing from 0.132 at low to 0.212 at high openness. These findings indicate that art education participation is associated with self-actualization partly through students' strengthened beliefs in their own creative capabilities, particularly among students high in openness to experience. Because the design was cross-sectional, these findings are framed as potential applications that require experimental or longitudinal evaluation rather than as established intervention guidance: universities may consider structured aesthetic education experiences as one resource for supporting students' eudaimonic wellbeing.

## 1 Introduction

Self-actualization, understood in the humanistic tradition as the full realization of one's talents, capacities, and potentialities, occupies a central place in theories of healthy psychological functioning (Maslow, 1943, 1968; Maunz and Glaser, 2023). Contemporary eudaimonic perspectives have carried this idea forward, defining wellbeing not merely as pleasure or life satisfaction but as the degree to which individuals live in accordance with their daimon, that is, their true self and personal potentials (Ryan and Deci, 2001; Ryff, 1989, 2023; Waterman, 1993). Although these perspectives differ in emphasis, they converge on the view that wellbeing involves the actualization of personal potentials; in the present research, self-actualization is treated within this shared humanistic-eudaimonic tradition, with a focus on its global, Maslow-style assessment rather than on the multidimensional profile captured by scales such as the Psychological Wellbeing scales (Ryff and Singer, 2008; see Margolis et al., 2022, for a recent measurement approach). The construct remains topical in contemporary research, which has examined its work-related antecedents (Maunz and Glaser, 2023) and reappraised Maslow's legacy for current psychology (Schulz and Cornejo, 2026). Self-actualization is also conceptually distinct from neighboring eudaimonic constructs: flourishing (Diener et al., 2010; Mostert et al., 2023), meaning in life (Steger et al., 2006; Chmiel and Kroplewski, 2025), psychological wellbeing (Ryff, 1989, 2023), and personal growth initiative (Jiao et al., 2024) capture, respectively, positive functioning across domains, purpose and significance, a multidimensional profile of positive functioning, and self-initiated growth behavior, whereas the present study targets the global, Maslow-based assessment of self-actualization. For college students, the transition to higher education is a formative period during which identity is consolidated and enduring orientations toward growth are established. Universities therefore function not only as sites of knowledge transmission but as meaningful developmental contexts that can either enable or constrain students' movement toward self-actualization. Understanding which educational experiences actually nourish this movement is thus an important task for educational and positive psychology.

Art education has long been proposed as one such experience. Proponents have argued that sustained engagement with the arts cultivates imagination, emotional depth, and the capacity for self-expression, qualities closely aligned with self-actualization (Winner and Hetland, 2000; Winner et al., 2013; Han and Lu, 2025). Empirically, a growing body of research links arts engagement with indicators of psychological wellbeing: a WHO-commissioned scoping review synthesized evidence from more than 3,000 studies suggesting that arts participation is associated with improved mental health and social cohesion across the life course (Fancourt and Finn, 2019; Bone et al., 2023; Crealey et al., 2023; Quinn et al., 2025), and qualitative work has identified meaning-making, identity work, and self-development as key mechanisms through which the arts benefit health (Davies et al., 2014; O'Donnell et al., 2023). In the specific domain of education, music learning has been associated with motivation, self-perceptions, and social skills among children and adolescents (Hallam, 2010; see Ding, 2026, for recent evidence on music learning and creative self-efficacy). Most of this evidence, however, concerns arts engagement in general, such as community-based arts participation, leisure arts activities, or arts-specific training, whereas the present study focuses on art education participation, namely students' structured involvement in the curricular and co-curricular art programs (public art courses, art electives, and campus arts activities) provided by their university; we draw on the broader arts engagement literature for mechanism hypotheses while treating the two constructs as related but not identical. In China, aesthetic education (meiyu) has been elevated in national education policy in recent years (Li et al., 2023), and Chinese universities have substantially expanded public art courses, art electives, and campus arts activities. Whether and how this expanding exposure to art education translates into students' self-actualization, however, remains largely an open question. Recent work in Chinese higher education indicates that aesthetic experience and art practice can support students' emotions, creativity, and self-identity (Han and Lu, 2025; Song et al., 2024), but has not yet examined self-actualization as an outcome, as most existing evidence comes from Western, school-age, or arts-specific samples rather than from general populations of higher education students.

A promising mechanism is creative self-efficacy, defined as the belief in one's ability to produce creative outcomes (Tierney and Farmer, 2002; Beghetto and Karwowski, 2023). According to self-efficacy theory, mastery experiences are the most powerful source of efficacy beliefs, and efficacy beliefs in one domain generalize to related domains of functioning (Bandura, 1997; Ding, 2026). Art education provides precisely such mastery experiences: studio work, ensemble performance, and creative projects offer frequent, structured opportunities to generate ideas, solve open-ended problems, and receive feedback, all of which can consolidate students' confidence in their creative capabilities. Correlational and intervention studies support this reasoning, showing that creative self-efficacy is nurtured by learning experiences characterized by autonomy and encouragement (Beghetto, 2006; Tierney and Farmer, 2002; Jaussi et al., 2007; Beghetto and Karwowski, 2023); recent research confirms that creative coursework and structured creative problem-solving raise creative self-efficacy among university students (Lee and Portillo, 2022; Snyder et al., 2025; Stolz et al., 2022) and that these beliefs evolve across the undergraduate years (Álvarez-Huerta et al., 2025). It also develops in dynamic interplay with creative identity and creative achievement across adolescence and young adulthood (Beghetto and Karwowski, 2023; Karwowski and Barbot, 2016). Creative self-efficacy, in turn, is a plausible antecedent of self-actualization: a student who trusts in his or her capacity to create is more likely to attempt challenging goals, persist through difficulty, and ultimately express latent potentialities, which is the essence of Maslow'Maslow, 1968 conceptualization (see also Maunz and Glaser, 2023). Emerging evidence is consistent with this reasoning: diary studies show that young adults report higher flourishing on days when they engage in creative activity (Conner et al., 2018; Smith et al., 2022). Recent evidence further shows that creative self-efficacy mediates the link between personality and mental wellbeing in university students (Fino and Sun, 2022) and is increasingly assessed alongside wellbeing outcomes (Holinger and Kaufman, 2024), and eudaimonic theory explicitly identifies self-realization through creative and exploratory activity as a core component of positive functioning (Ryff, 2023; Ryff and Singer, 2008). Positive emotions experienced during creative engagement may further broaden students' thought–action repertoires and build enduring personal resources (Csikszentmihalyi, 1996; Fredrickson, 2001; Mao et al., 2024). Creative self-efficacy may therefore operate as the psychological bridge through which art education participation promotes self-actualization.

The strength of this bridge, however, is unlikely to be uniform across students. Openness to experience, the Big Five dimension reflecting intellectual curiosity, aesthetic sensitivity, and receptiveness to novel ideas, is consistently identified as the strongest personality correlate of creativity and creative self-beliefs (McCrae, 1987; Si et al., 2025; Silvia et al., 2009) and is also positively associated with wellbeing (Anglim et al., 2020; Joshanloo, 2023), a link that also holds among college students in China (Jin et al., 2026). Students high in openness engage with art experiences more deeply: they are more likely to immerse themselves in aesthetic activities, elaborate on the ideas they encounter, and integrate these experiences into their self-concept, thereby extracting stronger mastery signals from the same participation (Csikszentmihalyi, 1996; Karwowski and Barbot, 2016; Si et al., 2025). By contrast, students low in openness may participate superficially, deriving little in the way of creative confidence. Openness to experience may thus moderate the first stage of the hypothesized pathway, strengthening the link from art education participation to creative self-efficacy, and, consequently, conditioning the indirect effect of art education participation on self-actualization. It should be acknowledged that openness is also associated with the propensity to seek out art experiences in the first place; the hypothesized moderation is therefore framed as a person–environment question of who converts participation into creative confidence more readily, while recognizing that selection and amplification processes may be intertwined (see Discussion).

The present study addressed these questions with a sample of Chinese college students, contributing to the literature in three ways. First, it extends arts and wellbeing research, which has concentrated on general wellbeing outcomes such as life satisfaction and affect, to self-actualization as a distinctly eudaimonic outcome. Second, it tests a conditional process model that specifies not only whether art education participation relates to self-actualization but how (through creative self-efficacy) and for whom (as a function of openness), thereby moving beyond simple main-effect questions (Hayes, 2022; Preacher et al., 2007). Third, by situating art education as a university resource for personal growth, the study speaks directly to the aim of this Research Topic of examining how meaningful educational contexts serve as resources for promoting wellbeing across the life course. Recent reviews have called for research that specifies the mechanisms and boundary conditions linking arts education to eudaimonic outcomes rather than documenting average associations (Vella-Brodrick et al., 2025), and the present model responds to that call. Three hypotheses were formulated. Consistent with prior evidence (Bone et al., 2023; Fancourt and Finn, 2019; Quinn et al., 2025; Winner et al., 2013), we hypothesized that art education participation would be positively associated with self-actualization (H1). Drawing on self-efficacy theory (Bandura, 1997; Ding, 2026), we hypothesized that creative self-efficacy would mediate this association (H2). Finally, based on personality evidence on openness and creativity (McCrae, 1987; Si et al., 2025; Silvia et al., 2009), we hypothesized that openness to experience would moderate the effect of art education participation on creative self-efficacy, such that the indirect effect of art education participation on self-actualization would be stronger among students higher in openness (H3).

## 2 Materials and methods

### 2.1 Participants

Participants were 671 undergraduate students (368 women, 54.8%; 303 men, 45.2%) aged 17–24 years (*M* = 19.9, SD = 1.3) recruited through convenience cluster sampling, in which intact general education classes were invited to participate, from three universities in Shandong Province, China (a comprehensive university, a normal university, and a university of science and engineering), covering humanities, social sciences, science, engineering, and arts majors; data were collected between October and November 2024. An *a priori* power analysis conducted with G^*^Power 3.1 for multiple regression (five predictors, *f* ^2^ = 0.05, α = 0.05, power = 0.95) indicated a minimum required sample of approximately 220; the achieved sample exceeded this requirement and provided adequate precision for bootstrap estimation of conditional indirect effects (Cohen, 1988; Faul et al., 2007; Lakens, 2022). Of 720 questionnaires distributed, 49 were excluded because of incomplete responses on the analysis variables or patterned responding, yielding a valid response rate of 93.2%. All analyses were based on the 671 valid cases; questionnaires with any missing data on the analysis variables were excluded listwise.

### 2.2 Measures

#### 2.2.1 Art education participation

Art education participation was assessed with five items developed for this study on the basis of prior arts participation research (Davies et al., 2014; Fancourt and Finn, 2019; Winner et al., 2013; see also Thapa et al., 2024; Jones et al., 2025, for recent operationalizations) and the five art domains emphasized in Chinese university aesthetic education curricula. Each item asked how frequently the student participated in one of five art domains within university art education settings: music, visual arts, dance, drama, and film appreciation (e.g., “How often do you take part in music-related courses, clubs, or activities organized by your university?”). Items were rated on a five-point scale ranging from 1 (never) to 5 (very frequently) and averaged, with higher scores indicating more frequent art education participation. It is important to state what this measure does and does not capture. Participation is defined here as the self-reported frequency of behavioral involvement in the art education settings provided by the university (public art courses, art electives, arts clubs, and campus arts activities) across the five art domains; the items therefore index behavioral participation rather than subjective involvement, engagement quality, objectively verified attendance, or artistic achievement, and we deliberately avoid conflating participation with broader constructs such as arts engagement, which also encompasses the quality and mechanisms of involvement (Thapa et al., 2024; Jones et al., 2025). Because the instrument was developed for this study, its development and validation require careful reporting. Items were generated from the five art domains emphasized in Chinese university aesthetic education curricula, reviewed for content appropriateness by two professors of psychology and fine arts education, and are domain-general frequency items that are averaged into a single score; as evidence of construct validity, the scale correlated in the expected direction with creative self-efficacy (*r* = 0.52) and openness to experience (*r* = 0.19). Cronbach's alpha was 0.87 in the present sample (Cronbach, 1951); recent methodological work emphasizes that alpha is a lower-bound, sample-specific estimate whose interpretation benefits from explicit discussion of its limitations (Cho, 2024; Kalkbrenner, 2024; McNeish and Dumas, 2025). An exploratory factor analysis (principal axis factoring with promax rotation) supported a single-factor structure [Kaiser–Meyer–Olkin (KMO) measure of sampling adequacy = 0.83; one factor with an eigenvalue above 1 explaining 61.4% of the variance; item loadings ranging from 0.62 to 0.81]; the index was introduced by Kaiser (1974), and its behavior under sampling error and item categorization has recently been evaluated by Lee et al. (2024).

#### 2.2.2 Creative self-efficacy

Creative self-efficacy was measured with the four-item Creative Self-Efficacy Scale (Tierney and Farmer, 2002; for recent applications in higher education, see Lee and Portillo, 2022; Snyder et al., 2025). A sample item is “I have confidence in my ability to solve problems creatively.” Items were rated on a five-point scale from 1 (strongly disagree) to 5 (strongly agree) and averaged, with higher scores reflecting stronger creative self-efficacy beliefs. Cronbach's alpha was 0.89 in the present sample.

#### 2.2.3 Self-actualization

Self-actualization was assessed with the Short Index of Self-Actualization (SISA; Jones and Crandall, 1986; for recent validations, see Hajipour et al., 2025; Salehi et al., 2025), a 15-item abbreviated measure of self-actualization. The SISA operationalizes the global, Maslow-based conception of self-actualization: its items index self-acceptance, spontaneity, and inner-directedness, that is, the extent to which individuals accept themselves and direct their lives in accordance with their own values and potentials. This is deliberately narrower than neighboring eudaimonic constructs, namely the multidimensional profile of psychological wellbeing (Ryff, 1989, 2023), positive functioning across domains indexed by flourishing measures (Diener et al., 2010; Mostert et al., 2023), purpose and significance (meaning in life; Steger et al., 2006; Chmiel and Kroplewski, 2025), and self-initiated growth behavior (personal growth initiative; Jiao et al., 2024). Because self-actualization is a broad and historically contested construct, a global measure was chosen to align with the humanistic tradition that frames eudaimonic wellbeing as the realization of personal potentials. A sample item is “I do not feel ashamed of any of my emotions.” Items were rated on a four-point scale from 1 (disagree) to 4 (agree); reverse-scored items were recoded, and item scores were averaged so that higher scores indicated greater self-actualization. Recent psychometric evaluations of the SISA in adult samples support its reliability and factor structure (Hajipour et al., 2025; Salehi et al., 2025). Cronbach's alpha was 0.91 in the present sample.

#### 2.2.4 Openness to experience

Openness to experience was measured with the 12-item openness domain of the Big Five Inventory-2 (BFI-2; Soto and John, 2017; for recent reliability evidence and a Chinese validation, see Husain et al., 2025; Shuai et al., 2026). A sample item is “I am curious about many different things.” Items were rated on a five-point scale from 1 (disagree strongly) to 5 (agree strongly) and averaged, with higher scores indicating greater openness. Cronbach's alpha was 0.82 in the present sample.

#### 2.2.5 Translation procedure

The English-language instruments were translated into Chinese and back-translated into English by two bilingual researchers independently; discrepancies were resolved through discussion to ensure semantic equivalence. The item content of all scales was reviewed by two professors of psychology and fine arts education to confirm appropriateness for the Chinese university context.

### 2.3 Procedure

Data were collected during the fall semester of the 2024–2025 academic year. Trained research assistants administered the paper-and-pencil questionnaire in regular classroom settings according to a standardized protocol. Participation was voluntary and anonymous; students provided written informed consent and were informed that they could withdraw at any time without penalty. Anonymity and confidentiality were emphasized in the oral instructions in order to reduce socially desirable responding (Crowne and Marlowe, 1960; Rickwood and Coleman-Rose, 2023). Self-report was considered appropriate because the constructs of interest concern students' own perceptions of their participation, creative confidence, and personal growth, which are not directly observable; at the same time, the questionnaire order was fixed rather than randomized and no social desirability scale was administered, which are acknowledged as limitations of the procedural design. The questionnaire battery took approximately 15–20 min to complete. The study was approved by the ethics committee of the authors' institution and was conducted in accordance with the Declaration of Helsinki.

### 2.4 Data analysis

Analyses were performed using SPSS 26 and the PROCESS macro (version 4.1; Hayes, 2022). Preliminary analyses included descriptive statistics, internal consistency estimates, and Pearson correlations. To evaluate the hypothesized conditional process model, PROCESS Model 7 was estimated with art education participation as the predictor, creative self-efficacy as the mediator, self-actualization as the outcome, and openness to experience as the moderator of the predictor–mediator path, with sex and age entered as covariates; sex and age were retained because both were associated with at least one study variable in preliminary analyses. Art education participation and openness to experience were mean-centered before the interaction term was computed, and variance inflation factors were all below 1.9 (range = 1.14–1.86), indicating no problematic multicollinearity. Before model estimation, all variables were screened for distributional shape; skewness and kurtosis values fell within ±1 for all study variables, supporting the use of the planned parametric analyses. Indirect and conditional indirect effects were estimated with 5,000 bootstrap resamples, and bias-corrected 95% confidence intervals were used; effects are significant when the confidence interval excludes zero (Hayes, 2022; Hayes and Rockwood, 2017; MacKinnon et al., 2004; Preacher et al., 2007). The index of moderated mediation, the product of the interaction coefficient and the mediator–outcome coefficient, was used to test H3 (Hayes, 2022). Because all measures were self-reported, Harman's single-factor test was conducted as an initial check of common method variance; the first unrotated factor accounted for 28.6% of the total variance, below the conventional 40% threshold, which provides preliminary evidence against substantial common method variance (Podsakoff et al., 2003, 2024; Spector, 2006). This test is a coarse diagnostic, however, and cannot establish the absence of common method variance (Manata and Boster, 2024); more informative procedures include unmeasured latent method constructs and marker variables (Ding et al., 2023), and the possibility of residual method effects is therefore revisited as a limitation. Statistical significance was set at *p* < 0.05 (two-tailed).

## 3 Results

### 3.1 Preliminary analyses

Descriptive statistics, internal consistency coefficients, and correlations among study variables are presented in Table 1. On average, students reported moderate levels of art education participation (*M* = 3.31, SD = 0.74), creative self-efficacy (*M* = 3.58, SD = 0.71), and openness to experience (*M* = 3.42, SD = 0.58), and a somewhat lower mean level of self-actualization (*M* = 2.98, SD = 0.42) on its four-point scale. As expected, art education participation was positively correlated with creative self-efficacy (*r* = 0.52, *p* < 0.001) and self-actualization (*r* = 0.38, *p* < 0.001); creative self-efficacy was positively correlated with self-actualization (*r* = 0.54, *p* < 0.001); and openness to experience was positively correlated with creative self-efficacy (*r* = 0.31, *p* < 0.001), self-actualization (*r* = 0.24, *p* < 0.001), and art education participation (*r* = 0.19, *p* < 0.001). These patterns provided initial support for the hypothesized model.

**Table 1:** Descriptive statistics, internal consistency, and correlations among study variables (*N* = 671).

Variable	*M*	SD	α	1	2	3	4
1. Art education participation	3.31	0.74	0.87	—			
2. Creative self-efficacy	3.58	0.71	0.89	0.52	—		
3. Self-actualization	2.98	0.42	0.91	0.38	0.54	—	
4. Openness to experience	3.42	0.58	0.82	0.19	0.31	0.24	—

α = Cronbach's alpha coefficient. All correlations were significant at *p* < 0.001. Art education participation, creative self-efficacy, and openness to experience were rated on five-point scales; self-actualization was rated on a four-point scale.

### 3.2 Testing the moderated mediation model

The conditional process model (PROCESS Model 7) explained a substantial proportion of variance in both creative self-efficacy (*R*^2^ = 0.31) and self-actualization (*R*^2^ = 0.36). As shown in Table 2, art education participation significantly and positively predicted creative self-efficacy (*a*_1_ = 0.39, SE = 0.043, *t* = 9.07, *p* < 0.001), supporting the first stage of the mediation hypothesis, and openness to experience was likewise a positive predictor of creative self-efficacy (*a*_2_ = 0.27, SE = 0.048, *t* = 5.63, *p* < 0.001). Critically, the interaction between art education participation and openness to experience significantly predicted creative self-efficacy (*a*_3_ = 0.09, SE = 0.032, *t* = 2.81, *p* = 0.005). Simple-slope analyses indicated that the slope of art education participation on creative self-efficacy was 0.30 (SE = 0.05) at one standard deviation below the mean of openness and 0.48 (SE = 0.06) at one standard deviation above the mean, confirming that participation translated into creative confidence more strongly among more open students. In the outcome model, creative self-efficacy significantly predicted self-actualization (*b* = 0.44, SE = 0.042, *t* = 10.48, *p* < 0.001), and art education participation retained a significant direct effect (*c*′ = 0.19, SE = 0.034, *t* = 5.59, *p* < 0.001), indicating partial mediation. The total effect of art education participation on self-actualization was significant (*c* = 0.36, SE = 0.033, *t* = 10.91, *p* < 0.001).

**Table 2:** Conditional process model results for creative self-efficacy and self-actualization (*N* = 671).

Model and predictor	*B*	SE	*t*	*p*
Mediator model: creative self-efficacy (*R*^2^ = 0.31)				
Constant	0.92	0.11	8.36	< 0.001
Art education participation (a_1_)	0.39	0.043	9.07	< 0.001
Openness to experience (a_2_)	0.27	0.048	5.63	< 0.001
Art education × openness (a_3_)	0.09	0.032	2.81	0.005
Sex (woman)	−0.04	0.050	−0.80	0.424
Age	0.02	0.025	0.80	0.424
Outcome model: self-actualization (*R*^2^ = 0.36)				
Constant	0.71	0.12	5.92	< 0.001
Creative self-efficacy (b)	0.44	0.042	10.48	< 0.001
Art education participation (c′)	0.19	0.034	5.59	< 0.001
Sex (woman)	−0.03	0.041	−0.73	0.465
Age	0.01	0.020	0.50	0.617
Total effect of art education participation (c)	0.36	0.033	10.91	< 0.001

Unstandardized coefficients are reported. Sex was coded 0 = man, 1 = woman; age was in years. Both models controlled for sex and age. The total effect model included art education participation and the covariates only.

### 3.3 Conditional indirect effects

The indirect effect of art education participation on self-actualization through creative self-efficacy, computed at the mean of openness to experience, was 0.172 [95% CI (0.132, 0.214)], accounting for 47.8% of the total effect. As shown in Table 3, the conditional indirect effect was significant at all three levels of openness to experience but increased monotonically from 0.132 [95% CI (0.092, 0.175)] at one standard deviation below the mean, to 0.172 [95% CI (0.132, 0.214)] at the mean, to 0.212 [95% CI (0.163, 0.263)] at one standard deviation above the mean. The index of moderated mediation was significant [0.040, 95% CI (0.008, 0.076)], indicating that the indirect effect of art education participation on self-actualization became stronger as openness to experience increased. Thus, H1, H2, and H3 were all supported. The conceptual model with unstandardized path coefficients is displayed in Figure 1.

**Table 3:** Conditional indirect effects of art education participation on self-actualization through creative self-efficacy at levels of openness to experience (*N* = 671).

Openness to experience	Value	Indirect effect	Boot SE	95% CI
M – 1 SD	2.84	0.132	0.021	[0.092, 0.175]
Mean	3.42	0.172	0.021	[0.132, 0.214]
M + 1 SD	4.00	0.212	0.025	[0.163, 0.263]
Index of moderated mediation	—	0.040	0.017	[0.008, 0.076]
Proportion of total effect mediated (at mean)	—	47.8%	—	—

Indirect effects were estimated with 5,000 bootstrap resamples; bias-corrected 95% confidence intervals are reported, and effects are significant when the confidence interval excludes zero. The index of moderated mediation is the product of the art education × openness interaction coefficient and the creative self-efficacy → self-actualization coefficient.

**Figure 1:**
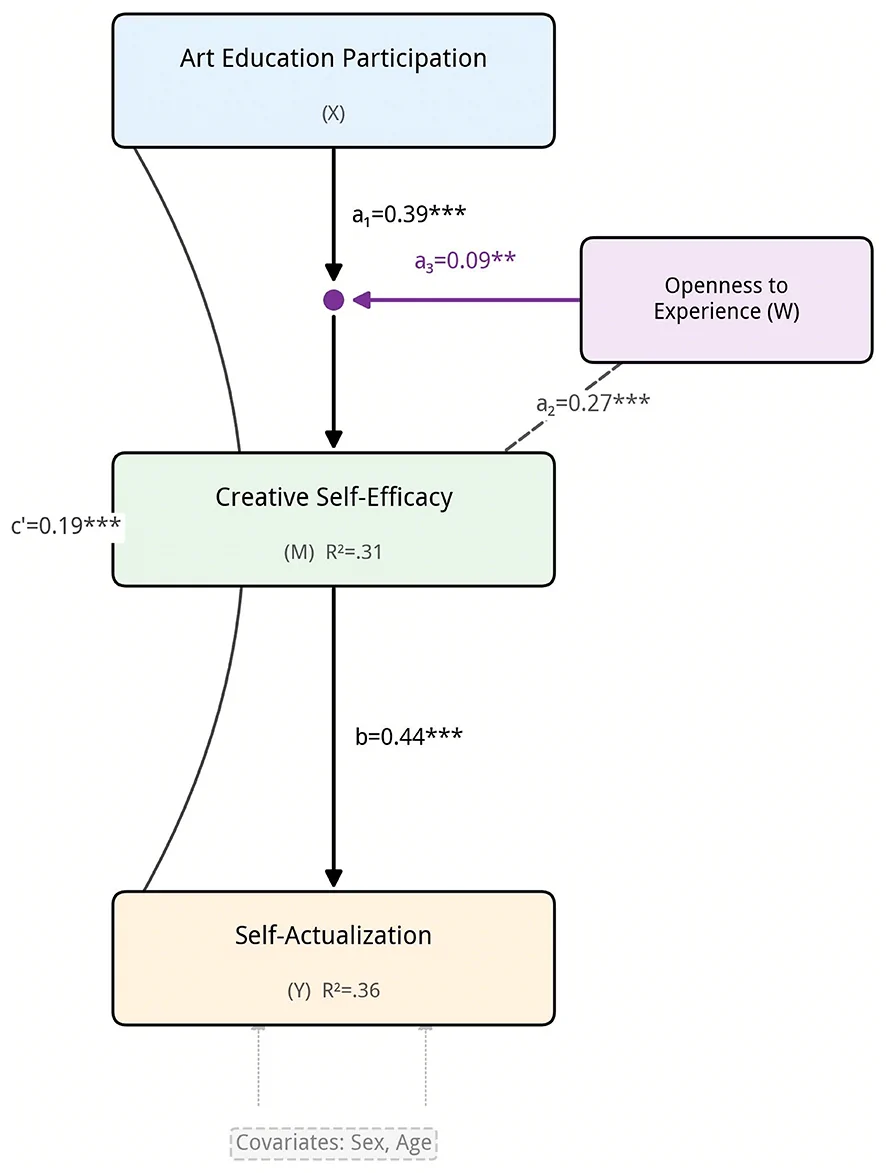
Conditional process model of the association between art education participation and self-actualization among Chinese college students (*N* = 671). Unstandardized coefficients are shown. Art education participation predicted creative self-efficacy (a_1_ = 0.39, *p* < 0.001), openness to experience predicted creative self-efficacy (a_2_ = 0.27, *p* < 0.001), and the art education × openness interaction predicted creative self-efficacy (a_3_ = 0.09, *p* = 0.005). Creative self-efficacy predicted self-actualization (b = 0.44, *p* < 0.001), and the direct effect of art education participation on self-actualization remained significant (*c*′ = 0.19, *p* < 0.001; total effect *c* = 0.36, *p* < 0.001). The model controlled for sex and age; *R*^2^ = 0.31 for creative self-efficacy and *R*^2^ = 0.36 for self-actualization. The figure was created by the authors. ^**^p < 0.01 and ^***^p < 0.001.

## 4 Discussion

This study examined whether and how art education participation relates to self-actualization among Chinese college students. Three main findings emerged. First, art education participation was positively associated with self-actualization. Second, creative self-efficacy partially mediated this association, accounting for nearly half of the total effect. Third, openness to experience moderated the first stage of the indirect pathway, such that the pathway was stronger among students higher in openness. Together, these findings portray university art education as a meaningful context that can nourish eudaimonic growth, and they identify who is most likely to benefit from it.

The positive association between art education participation and self-actualization (H1) extends the arts and wellbeing literature from hedonic outcomes, such as positive affect and life satisfaction, to a distinctly eudaimonic one. This convergence is consistent with evidence that arts engagement supports mental health and social wellbeing across the life course (Fancourt and Finn, 2019; Davies et al., 2014; Bone et al., 2023; Quinn et al., 2025) and with long-standing claims that the arts cultivate qualities central to self-actualization, including openness to experience, spontaneity, and acceptance of oneself (Maslow, 1968; Winner and Hetland, 2000; Winner et al., 2013). The result also resonates with eudaimonic theorizing, which locates wellbeing in the actualization of personal potentials rather than in the maximization of pleasure (Ryan and Deci, 2001; Ryff, 1989, 2023; Waterman, 1993). In the Chinese higher education context, where aesthetic education has been expanded as part of a broader policy emphasis on whole-person development (Li et al., 2023), and where recent studies show that aesthetic experience and art practice support students' affective, creative, and self-identity development (Song et al., 2024; Han and Lu, 2025), the present findings provide empirical grounding for the view that art education is not merely an ornament to the curriculum but a resource for students' personal growth.

The mediation finding (H2) supports the proposed self-efficacy mechanism. Art education settings supply the ingredients that self-efficacy theory identifies as most potent for belief formation: mastery experiences gained through completing creative work, vicarious experiences provided by peers and instructors, and verbal persuasion through feedback and critique (Bandura, 1997; Beghetto and Karwowski, 2023). Through repeated creative accomplishment, students appear to develop a generalized confidence in their creative capabilities (Tierney and Farmer, 2002; Beghetto, 2006; Jaussi et al., 2007; Snyder et al., 2025), and creative activity itself has been found to accompany higher flourishing in daily life (Conner et al., 2018; Smith et al., 2022). The present indirect effect is consistent with recent evidence that creative self-efficacy carries the influence of dispositional and contextual antecedents to wellbeing (Fino and Sun, 2022) and that creativity-related self-beliefs and wellbeing are closely intertwined in student samples (Holinger and Kaufman, 2024). This confidence in turn disposes them to pursue challenging goals, persist in the face of difficulty, and express their potentialities, the behavioral signature of self-actualization. The substantial proportion of the total effect transmitted through creative self-efficacy (47.8%) suggests that strengthening students' creative confidence may be among the more consequential psychological products of art education. The direct effect nonetheless remained significant, indicating that other mechanisms, such as emotional regulation, meaning-making, or flow experiences (Csikszentmihalyi, 1996; Fredrickson, 2001; Mao et al., 2024), likely operate in parallel; identifying these additional pathways is a task for future research.

The moderated mediation finding (H3) adds a boundary condition to this account. Openness to experience amplified the effect of art education participation on creative self-efficacy, and correspondingly the indirect effect on self-actualization increased from 0.132 at low to 0.212 at high openness. Students high in openness are intrinsically drawn to aesthetic stimulation, engage with art experiences more deeply, and elaborate the ideas they encounter more richly, thereby converting the same dose of participation into stronger mastery signals (Beghetto and Karwowski, 2023; Csikszentmihalyi, 1996; Karwowski and Barbot, 2016). This pattern is consistent with evidence that openness is the strongest personality correlate of creativity and creative self-beliefs (McCrae, 1987; Si et al., 2025; Silvia et al., 2009) and with meta-analytic findings linking openness to wellbeing (Anglim et al., 2020; Joshanloo, 2023), and with recent evidence that openness conditions the wellbeing benefits of art-related aesthetic experience among college students (Jin et al., 2026). From a person–environment fit perspective, the finding suggests that the benefits of art education are contingent on the match between the affordances of the environment and the dispositions of the person: aesthetic environments appear to function best as growth contexts for those prepared to receive them. Two caveats qualify this interpretation. First, the amplification account offered here is *post hoc*; the interaction pattern is consistent with it but was not tested against competing explanations. Second, because openness is also associated with seeking out art experiences and with deeper engagement once present, the moderation may partly reflect differences in the quality or intensity of participation rather than a purely psychological amplification of otherwise identical experiences; research measuring engagement quality directly is needed to separate these possibilities.

Viewed against research published since 2022, these findings speak to three ongoing conversations. First, population-level studies have established that arts engagement predicts subsequent flourishing and better mental health in longitudinal designs (Bone et al., 2023; Fluharty et al., 2023; Quinn et al., 2025), yet they have largely remained silent about the educational settings and psychological mechanisms that generate such effects. The present results specify one plausible mechanism, creative self-efficacy, and locate it in a setting, university art education, that is amenable to institutional design. Second, recent investigations of aesthetic education in China have documented affective and creative benefits of aesthetic experience and art practice (Song et al., 2024; Han and Lu, 2025) and resonate with policy analyses of aesthetic education (Li et al., 2023); extending that work, the present findings indicate that the benefits are not confined to momentary affect or creative self-identity but extend to self-actualization as a eudaimonic outcome. Third, current personality research emphasizes both the correlates of wellbeing (Joshanloo, 2023) and the moderating role of openness in aesthetic and creative processes (Si et al., 2025; Jin et al., 2026). The moderation result is consistent with these accounts while also qualifying them: rather than treating openness as a static predictor, it identifies openness as a boundary condition that determines how much creative confidence is extracted from the same educational exposure.

These findings carry potential implications for higher education, although they require evaluation before they are treated as recommendations: the cross-sectional design does not establish that introducing art education increases self-actualization, so the points below are framed as hypotheses for experimental or longitudinal testing rather than as evidence-based intervention guidance. First, they suggest that universities may count art education among their instruments for promoting eudaimonic wellbeing, a goal that aligns with growing calls to treat student flourishing as a core institutional mission rather than an incidental by-product of study (Vella-Brodrick et al., 2025). Second, because creative self-efficacy carried nearly half of the total effect, art courses and activities could be structured to generate visible mastery experiences, for example by sequencing assignments from achievable to challenging, providing encouraging and specific feedback, and showcasing student creative work (Tripon, 2021; Vella-Brodrick et al., 2025). Third, the moderation result cautions against one-size-fits-all provision. Students low in openness, who currently derive the least benefit, may need structured introductions to the arts, guided appreciation experiences, and gradual exposure to reduce the threshold for engagement, whereas students high in openness can be challenged with open-ended creative projects. Observing which students gravitate toward aesthetic activities, and remaining alert to differences in openness when designing programming, may help student affairs professionals match provision to students more effectively, without treating openness as a fixed gatekeeper to arts education.

Three contributions appear specific to the present evidence base. First, whereas research published since 2022 has mainly linked arts engagement to hedonic or broad wellbeing outcomes (Bone et al., 2023; Quinn et al., 2025) or to creative self-beliefs within a single artistic domain (Lee and Portillo, 2022; Ding, 2026), this study connects institutionally provided art education across five art domains to a distinctively eudaimonic outcome, self-actualization, in a large sample of Chinese undergraduates. Second, it provides an empirical test of a conditional process model that recent reviews recommend but that has rarely been examined in this literature: the field has been urged to move beyond average effect sizes toward mechanisms and boundary conditions (Vella-Brodrick et al., 2025), and the present results identify creative self-efficacy as a mechanism and openness as a moderator of that mechanism. Third, it responds to calls to treat aesthetic education in Chinese universities as a psychological resource (Li et al., 2023; Han and Lu, 2025) by quantifying how much of the association between participation and self-actualization flows through a measurable, modifiable belief; the 47.8% mediated proportion provides a benchmark against which future intervention studies can be evaluated.

Several limitations qualify these conclusions, and each carries a recognizable source of bias. First, the cross-sectional design precludes causal inference; the bias at issue is the absence of temporal precedence and the possibility of reciprocal causation, which has long been documented for cross-sectional analyses of mediation (Maxwell and Cole, 2007) and remains the subject of current methodological debate (Barnett et al., 2023; Savitz and Wellenius, 2023). Although the hypothesized causal ordering is grounded in theory, alternative orderings, such as self-actualized students seeking out art education, cannot be ruled out, and longitudinal or experimental designs are needed. In addition, several potential confounds, including prior arts training background and family socioeconomic status, were not measured; the relevant bias is selection or omitted-variable bias, a general problem of non-experimental data (Heckman, 1979) that also arises in participation research, where those who take part differ systematically from those who do not (Hiratsuka, 2025). Second, all measures were self-reported, so common method variance and social desirability bias may inflate the observed associations: emphasizing anonymity can reduce but not eliminate socially desirable responding (Crowne and Marlowe, 1960; Rickwood and Coleman-Rose, 2023), and Harman's single-factor test has limited sensitivity (Podsakoff et al., 2003, 2024; Spector, 2006), with method variance continuing to be treated as a serious threat in current methodological commentary (Manata and Boster, 2024). Self-report also constrains the assessment of behavioral constructs such as participation frequency, as recent validity research on self-report measures documents (Vangsness et al., 2022). Third, the measure of art education participation was developed for this study; the associated bias is construct underrepresentation, that is, the risk that a measure fails to capture the full breadth of the intended construct (Cronbach and Meehl, 1955), and current work on arts engagement measurement shows how much conceptual ground lies beyond participation frequency alone (Jones et al., 2025; Thapa et al., 2024). Although the scale showed acceptable reliability and a coherent single-factor structure, further validation of its factor structure and criterion validity in independent samples is warranted. Fourth, the sample was drawn from three universities in a single province through convenience sampling; the resulting generalizability bias is well documented for narrow, non-representative samples (Henrich et al., 2010) and continues to be discussed in current sampling research (Doebel and Frank, 2024; Novielli et al., 2025). Finally, self-actualization was measured with a brief global index (Jones and Crandall, 1986); although recent psychometric evaluations support the SISA (Hajipour et al., 2025; Salehi et al., 2025), a short global measure cannot capture the full breadth of eudaimonic functioning, and multi-dimensional assessments would allow a finer-grained analysis of which facets of self-actualization are most responsive to art education.

Future research should address these limitations directly. Longitudinal designs with at least three waves, ideally analyzed with cross-lagged panel or random-intercept cross-lagged models, would allow the ordering of participation, creative self-efficacy, and self-actualization to be tested rather than assumed (Barnett et al., 2023; Maxwell and Cole, 2007; Savitz and Wellenius, 2023). Experience-sampling or daily diary designs would capture how creative confidence and self-actualization fluctuate around specific art education experiences (Conner et al., 2018; Smith et al., 2022), and experimental or quasi-experimental evaluations of structured art courses would provide the evidence required before intervention recommendations are issued (Tripon, 2021; Vella-Brodrick et al., 2025). Measurement research is also needed: validated multi-domain instruments that separate behavioral participation from subjective engagement would clarify which component drives the effect (Jones et al., 2025; Thapa et al., 2024), and multi-source or behavioral indicators of creative achievement would reduce reliance on self-report (Manata and Boster, 2024). Finally, testing the model with more representative samples and in other cultural and institutional contexts (Doebel and Frank, 2024) would establish the boundaries of the account offered here.

## 5 Conclusion

This study set out to examine whether art education participation is positively associated with self-actualization among Chinese college students (H1), whether creative self-efficacy mediates that association (H2), and whether openness to experience moderates the first stage of the indirect pathway, such that the indirect effect is stronger among students higher in openness (H3). All three hypotheses were corroborated, although to different degrees. H1 was supported by a moderate positive association (*r* = 0.38) and a significant total effect (*c* = 0.36). H2 was supported by a significant indirect effect that accounted for 47.8% of the total effect; because the direct effect remained significant (*c*′ = 0.19), the corroboration is partial rather than full mediation. H3 was supported by a significant interaction between art education participation and openness (*a*_3_ = 0.09, *p* = 0.005) and by a significant index of moderated mediation [0.040, 95% CI (0.008, 0.076)], with the conditional indirect effect increasing from 0.132 at low openness to 0.212 at high openness. The corroboration is therefore strong but bounded: because the design was cross-sectional, the causal ordering implied by these hypotheses remains an inference rather than a demonstration. Within that constraint, the findings identify a modifiable educational experience, a tractable psychological mechanism, and a meaningful individual-differences boundary, and they portray university art education as one context in which students' eudaimonic development may be supported.

## Data Availability

The raw data supporting the conclusions of this article will be made available by the authors, without undue reservation.
